# Apoplast proteomic analysis reveals drought stress-responsive protein datasets in chilli (*Capsicum annuum* L.)

**DOI:** 10.1016/j.dib.2019.104041

**Published:** 2019-05-23

**Authors:** N. Jaswanthi, M.S.R. Krishna, U. Lakshmi Sahitya, P. Suneetha

**Affiliations:** a Department of Biotechnology, KLEF, Guntur, Andhra Pradesh, 522502, India; b Institute of Biotechnology, Prof. Jaya Shankar Telangana State Agricultural University, Hyderabad, 500030, India

**Keywords:** Antioxidant, Apoplast proteome, *Capsicum annuum,*, Drought, LC-MS

## Abstract

Drought is one of the major environmental constrains that limit plant performance worldwide. Plant apoplast which acts as connecting link between environment and plant protoplast carries multiple functions in plant metabolism and signalling. To investigate the drought induced changes in apoplast, proteome analysis in conjunction with antioxidant enzyme activity changes were studied in chilli (Capsicum annuum L). Drought induced apoplast proteome revealed augmented phenyl alanine ammonia lyase, peroxidase activities and reduced catalase activity. LC-MS analysis of apoplast proteome revealed differential expression of proteins under water stress conditions. Up-regulation of 43 protein species which encompass stress related proteins such as defensins, peroxidises, polygalaturonase inhibitor proteins, superoxide dismutase proteins were observed. Unlike control, twenty unique protein species were identified to be present in proteome of drought treated plants. Qualitative and quantitative changes in apoplast proteome emphasize the dynamics of plant apoplast and its role in drought stress. This work would provide insights into drought induced proteomic changes in apoplast and also would prove to be useful for protein phenotyping.

**Table:** Specifications table

Subject area	Plant biology
More specific subject area	Proteomics
Type of data	Tables, figures
How data was acquired	LC-MS Analysis is performed in 1290 Infinity UHPLC system, 1260 infinity Nano HPLC with Chip cube, 6550 iFunnel Q-TOFs (Agilent technologies, USA)
Data format	Raw and analysed data
Experimental factors	Elite chilli genotype (S-10) seeds were procured and seedlings were transplanted at 45DAS and allowed for acclimatization for 10 days. Then plants were subjected to drought at 100% and 40% FC for one week.
Experimental features	Apoplastic sample was extracted from the treated and control plant leaves through infiltration method using extraction buffer (0.1 M potassium phosphate buffer pH-7). Then phenyl alanine ammonia lyase, peroxidase and catalase activities and phenol content were estimated in the apoplastic fluid and proteomic analysis was done by using LC-MS analysis.
Data source location	Sophisticated Analytical Instrument Facility (SAIF), IIT Bombay, INDIA.
Data accessibility	Data is available with this publication

**Table:** 

**Value of the data** • Investigated data highlight the apoplastic changes in leaf proteome of chilli genotype (S- 10), which is valuable for researches working on drought stress tolerance. • Leaf apoplastic proteomic data along with enzyme activities of drought stressed plant was compared to that in control plants of chilli genotype. This suggests changes in protein regulation under drought conditions. • Increased levels of phenol and increased activities of peroxidase and catalase enzymes in leaf apoplast act as one of important factors for conferring drought tolerance which is an important value for crops growing in arid and semi-arid regions. • Analyzed LC-MS data revealed the proteomic changes that have occurred in chilli leaf apoplast during stress conditions is a valuable to researchers working on drought stress that effects the plant growth and development. • Present apoplastic LC-MS data and enzyme activity data provide information for identification of the candidate proteins and development of protein based markers which can be ultimately used by plant breeders and scientists in n chilli crop improvement.

## 1 Data

We present proteome data and enzyme activity data of leaf apoplast subjected to drought conditions. Elite chilli genotype (S-10) was subjected to two water regimes - 100% Field Capacity (Control) and 40% Field Capacity (Drought Treated). Malate Dehydrogenase (MDH) activity and was found to be 1% in treated and 0.4% in control apoplast samples. Malate Dehydrogenase activity is widely used as a specific marker to identify degree of cell membrane integrity and level of cytosolic contamination [1].

**Fig. 1:**
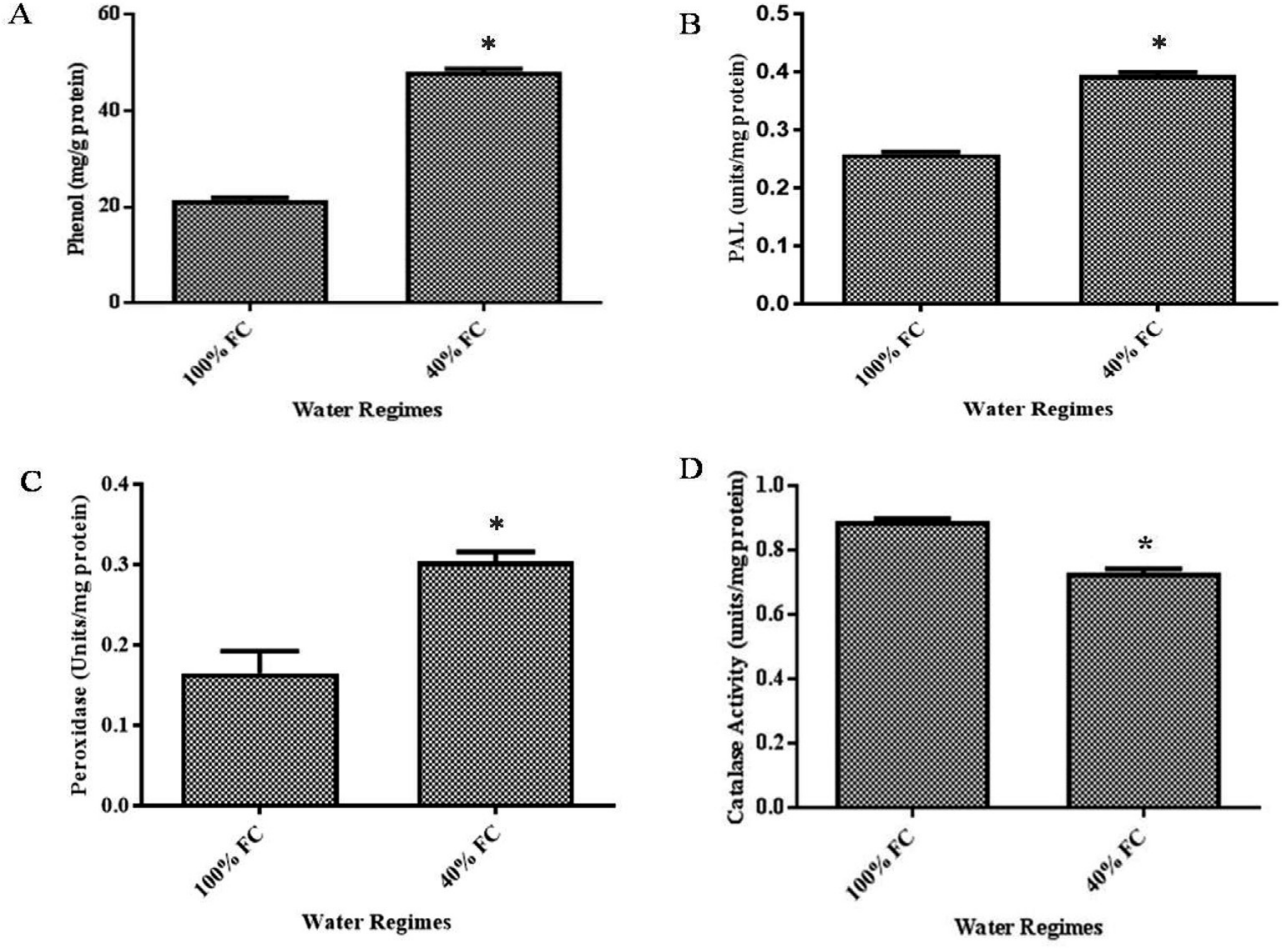
Changes in Phenol content (A), PAL activity (B), Peroxidase activity (C) and Catalase activity (D) under 100% and 40% FC. Values are represented as mean ± SD. * represents significant difference at P = 0.05.

In the present data, Fig. 1 represents effect of drought on contents of apoplast. Phenolic content in control (21.4 mg/g) and treated (48.02 mg/g) was represented in Fig. 1A. There observed an increase in phenolic content by 1.24 folds under drought. In *Vitis vinifera* there was an increased production of phenolics under abiotic stress conditions [2]. Phenylalanine Ammonia Lyase (PAL) activity was also increased in drought by 0.56 folds (Fig. 1B). Our results were in accordance with Gholizadeh [3]. The peroxidase activity in control and stressed samples were 0.16 and 0.30 units/mg protein respectively (Fig. 1C). Increased peroxidase activity in apoplast under stress conditions was also reported in wheat root cells [4], chilli leaves [5]. Data revealed a decrease by 17.9% in treated (0.73 units/mg protein) when compared to control (0.89 units/mg protein) (Fig. 1D). Drought induced decline in catalase activity was observed in wheat [6]. Statistical analysis was provided in supplementary File S1.

### 1.1 LC-MS analysis.

A total 208 protein species were identified from the LC-MS analysis of control and treated proteomes. Among 208 protein species, eight were of different origins such as, cytoplasm (5), ribosomal (2) and mitochondrial (1) origin due to cytoplasmic contamination of apoplast fluid. LC-MS proteome data of 208 protein species was provided in Supplementary Table S2.

**Fig. 2:**
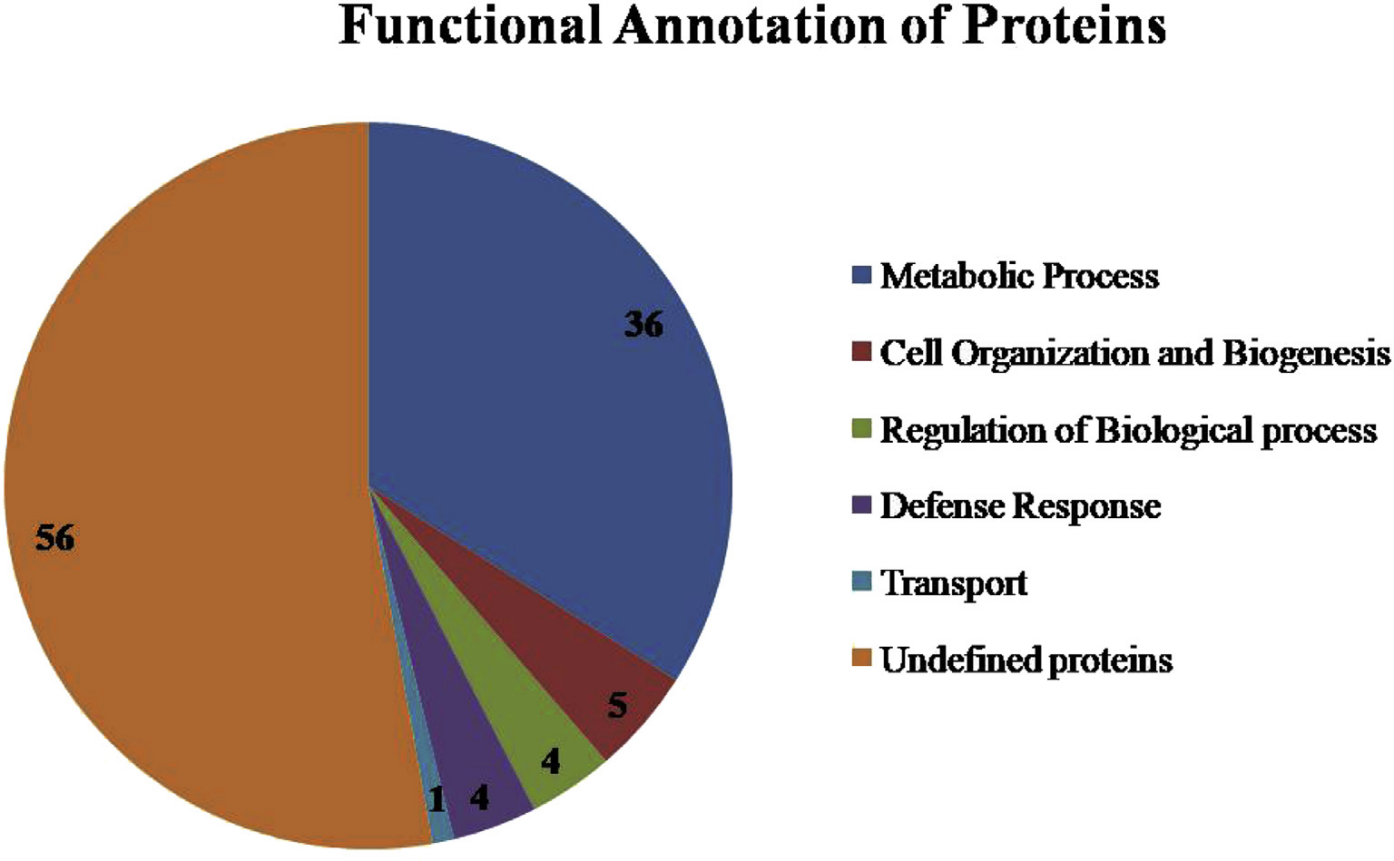
Functional Annotation of 106 proteins identified in chilli leaf apoplast.

Among the 208, 106 protein species were considered for further analysis, as they contain at least 2 unique peptides. Trentin et al. [7], also studied proteins with at least 2 unique peptides in *Arabidopsis thaliana* apoplast proteome. Based on their role in Biological process, 106 proteins were categorised into six groups (Fig. 2) *viz*., metabolic process (Table 1), cell organization and biogenesis (Table 2), regulation of biological process (Table 3), defense response and transport functions (Table 4) and undefined proteins (Table 5).

**Table 1:** Abundance change in protein species involved in metabolic process during the drought stress in chilli.

S.NO	Accession	Description	MW [kDa]/calc.pI	Abundance Ratio: (T/C)	S.NO	Accession	Description	MW [kDa]/calc.pI	Abundance Ratio: (T/C)
1	A0A1U8E5V7	beta-xylosidase	85.6/8.06	0.684	17	K4FXE7	Triose phosphate isomerase	27.1/5.99	100
2	A0A1U8G957	Peroxidase	34.4/8.19	2.095	18	A0A1U8HJ07	Malate dehydrogenase	36.1/8.9	0.059
3	A0A1U8FME6	Acidic endochitinase Q	27.6/7.12	1.304	19	A0A1U8GJW9	aspartyl protease	46.3/8	100
4	A0A1U8GIQ7	subtilisin-like protease	79/6.48	0.794	20	A0A1U8E7X6	subtilisin	82.9/6.35	0.245
5	A0A075VXE8	Uncharacterized protein	53.7/7.05	1.232	21	A0A1U8FZZ3	alpha-l-fucosidase	55.8/8.41	100
6	A0A1U8F4N5	glucanendo-1,3-beta-glucosidase	38.4/7.52	0.548	22	A0A1U8H921	acidic mammalian chitinase	42.4/8.81	0.99
7	A0A1U8FXF2	Acidic endochitinase pcht28	27.3/4.98	100	23	A0A1U8FRY3	Uncharacterized protein	27.4/5.96	0.613
8	A0A1U8ECS7	early nodulin	35.7/8.78	0.731	24	J1KTS6	ATP synthase subunit beta	54.1/5.06	0.424
9	A0A1U8HET9	ribonuclease MC	34.5/7.11	1.073	25	A0A1U8E6R9	basic 30 kDa endochitinase	34.6/6.81	100
10	A0A1U8H8B0	ribonuclease MC	35.7/7.23	1.377	26	A0A1U8FT89	zingipain-2-like	38.4/6.23	1.505
11	A0A1U8EQG4	acetylajmalan esterase	41.2/8.98	1.368	27	A0A1U8FA63	aspartic proteinase	46.4/7.91	0.088
12	B9VRK9	Peroxidase	34.9/9.2	0.674	28	A0A1U8FIT0	Uncharacterized protein	82.2/8.48	0.493
13	A0A1U8GZB5	Peroxidase	35/4.79		29	A0A1U8GJ92	subtilisin	81.8/5.9	0.694
14	A0A1U8GAI0	CO(2)-responsesecreted protease	81.2/5.94	0.321	30	A0A1U8GNT7	Alpha-amylase	48.1/6.16	0.284
15	A0A1U8DUD4	subtilisin	83.7/7.59	3.355	31	A0A1U8FYA1	Peroxidase	36.3/9.2	0.448
16	A0A1U8H5T7	Somatic embryogenesis receptor kinase	21.3/7.85	0.202	32	A0A1U8H994	reticuline oxidase	62.8/8.95	0.3
33	A0A1U8EYS7	Peptidylprolyl isomerase	23.6/8.41	100	35	A0A1U8GCP7	alpha-xylosidase	104.7/6.9	0.126
34	A0A1U8ELC6	Elongation factor 1-alpha	49.3/9.13	0.445	36	A0A1U8GSD0	Glucan endo-1,3-beta-glucosidase	52.9/5.97	100

**Table 2:** Proteins involved in cell organization and biogenesis identified by LC-MS analysis.

S.NO	Accession	Description	MW [kDa]/calc.pI	Abundance Ratio: (T/C)
1	A0A1U8F2I8	Pectin acetylesterase	44.4/8.43	1.83
2	A0A1U8F8D2	Pectinesterase	63/8.91	0.381
3	A0A1U8FU93	Pectinesterase	64.5/9	0.434
4	A0A1U8E7U4	heat shock cognate protein	71.2/5.22	0.242
5	A0A1U8FC85	Pectinesterase	60.1/7.25	100

**Table 3:** Drought induced change in protein abundance involved in regulation of biological process.

S.NO	Accession	Description	MW [kDa]/calc.pI	Abundance Ratio: (T/C)
1	A0A1U8G802	Germin-like protein	21.6/7.44	0.743
2	T1PZ85	Pin-II type proteinase inhibitor	28.7/5.81	100
3	Q4ZIQ4	Pin-II type proteinase inhibitor	28.5/6.44	100
4	A0A1U8E8X8	Miraculin	25/9.03	0.039

**Table 4:** Change in the abundance of defense response and transportation function related protein species during drought stress.

Defense response				
S.NO	Accession	Description	MW [kDa]/calc.pI	Abundance Ratio: (T/C)
1	A0A1U8H8C8	flower-specific defensin	9.5/8.24	1.794
2	A0A023JGE3	Stress-induced protein	10.2/5.97	0.907
3	A0A1U8HEV9	flower-specific defensin	12/7.09	100
4	A0A1U8H869	defensin-like protein	9.9/7.83	100

Transportation function				

1	A0A1U8E2G3	Non-specific lipid-transfer protein	13.3/8.41	0.541

**Table 5:** Undefined proteins obtained from LC-MS analysis.

S.NO	Accession	Description	MW [kDa]/calc.pI	Abundance Ratio: (T/C)	S.NO	Accession	Description	MW [kDa]/calc.pI	Abundance Ratio: (T/C)
1	A0A2G2XYJ9	probable carbohydrate esterase	29.5/8.72	0.719	17	A0A2G3AL78	Globulin	46.8/8.35	0.385
2	A0A2G2YI88	Beta-galactosidase	92.5/7.66	0.674	18	A0A2G2ZSR6	l-ascorbate oxidase homolog	59.8/9.03	0.971
3	A0A2G2YXJ9	aspartyl protease	52.4/8.54	0.623	19	A0A2G3A116	Antimicrobial protein	12.8/8.95	5.846
4	A0A1U8G2S5	Polygalacturonase inhibitor 1	36.7/8.27	0.659	20	A0A2G2YKI8	Non-specific lipid-transfer protein	13.7/8.95	1.976
5	A0A2G2YGW0	putative amidase	54.2/9.09	5.69	21	A0A1U8GHD2	neutral ceramidase	85.7/8.09	100
6	A0A2G2ZCU6	Miraculin	23.5/8.95	0.419	22	A0A2G2ZI14	Transketolase, chloroplastic	80.9/6.04	100
7	A0A2G2YGS4	Uncharacterized protein	53.7/8.63	100	23	A0A2G2YVL9	proline-rich protein	25.8/9.33	1.664
8	A0A1U8HA07	Auxin-binding protein	22/6.77	1.004	24	A0A2G2Z8Q7	Superoxide dismutase	28.2/8.28	0.609
9	A0A1U8E5C4	pathogenesis-related leaf protein	17.4/8.32	0.217	25	A0A2G2YHQ5	Ripening-related protein	28.8/5.69	0.275
10	A0A2G2Y9E7	aspartyl protease	47.9/8.34	1.212	26	A0A2G2ZD03	Miraculin	22.9/8.21	100
11	A0A2G3AJY5	Uncharacterized protein	56.5/6.05	0.78	27	A0A1U8EU88	uncharacterized protein	25.3/7.9	0.221
12	A0A2G2Y8V4	pathogenesis-related protein	28.5/7.94	100	28	E9JEC2	Epidermis-specific secreted glycoprotein	33.7/9.36	100
13	A0A1U8G7K7	thaumatin-like protein	24.2/8.18	1.138	29	A0A2G3AM91	Nucleoside-diphosphate kinase	16.3/6.79	100
14	A0A1U8E849	Basic secretory protease	25.3/8.53	1.022	30	A0A2G2Y4E8	Cysteine proteinase inhibitor	12.9/8.94	3.47
15	A0A1U8E4D3	desiccation-related protein	37.8/8.31	2.329	31	A0A2G2Y9I5	Uncharacterized protein	81.6/6.04	0.064
16	A0A1U8FKK0	protein trichome birefringence	46.3/9.04	3.586	32	A0A2G2Y352	Uncharacterized protein	26.9/9.79	0.335
33	A0A2G2ZG61	alpha-glucosidase	100.8/6.74	100	45	A0A2G2YKB1	Non-specific lipid-transfer protein	15.5/8.24	1.398
34	A0A2G2YYV2	Uncharacterized protein	48.7/9.25	1.455	46	A0A2G2ZW93	Non-specific lipid-transfer protein	12.8/8.66	0.425
35	A0A2G2XYL3	Expansin	28.2/7.99	1.126	47	A0A2G2V7A7	Uncharacterized protein	9.2/8.13	3.238
36	A0A2G3ADZ2	protein P21	25.1/6.81	0.527	48	A0A2G3AB80	Subtilisin	81.3/6.6	0.652
37	A0A2G2ZAL7	Non-specific lipid-transfer protein	15.5/8.94	1.177	49	A0A2G3AAY0	probably LRR receptor	51.8/8.81	0.975
38	D9IC46	Polygalacturonase-inhibiting proteins	29.8/9.13	1.856	50	A0A2G2ZC11	Alpha-mannosidase	116.9/6.58	0.479
39	A0A2G3A9X4	Endochitinase	37/9.26	0.641	51	A0A2G2XAU5	Endochitinase B	32.8/5.03	2.717
40	A0A2G3AA26	acidic endochitinase	27.8/9.14	0.56	52	A0A1U8FSR6	Ribulose bisphosphate carboxylase	20.5/8.13	0.825
41	A0A2G2YHG3	Carboxypeptidase	57.1/5.6	1.231	53	A0A1U8FR64	uncharacterized protein	54.9/8.1	0.637
42	A0A2G2YNI0	alpha-l-arabinofuranosidase	74/5.83	0.404	54	A0A2G2YU11	cysteine-rich repeat protein	26.8/7.01	0.325
43	A0A2G2Y3P2	Carboxypeptidase	55.4/7.08	0.375	55	A0A1U8DSA1	Uncharacterized protein	40.6/7.14	2.025
44	A0A059P572	Polygalacturonase inhibiting protein	38.9/8.87	0.382	56	A0A2G2YNT9	Glucan-endo-1,3-beta-glucosidase	37.9/9.03	0.609

**Fig. 3:**
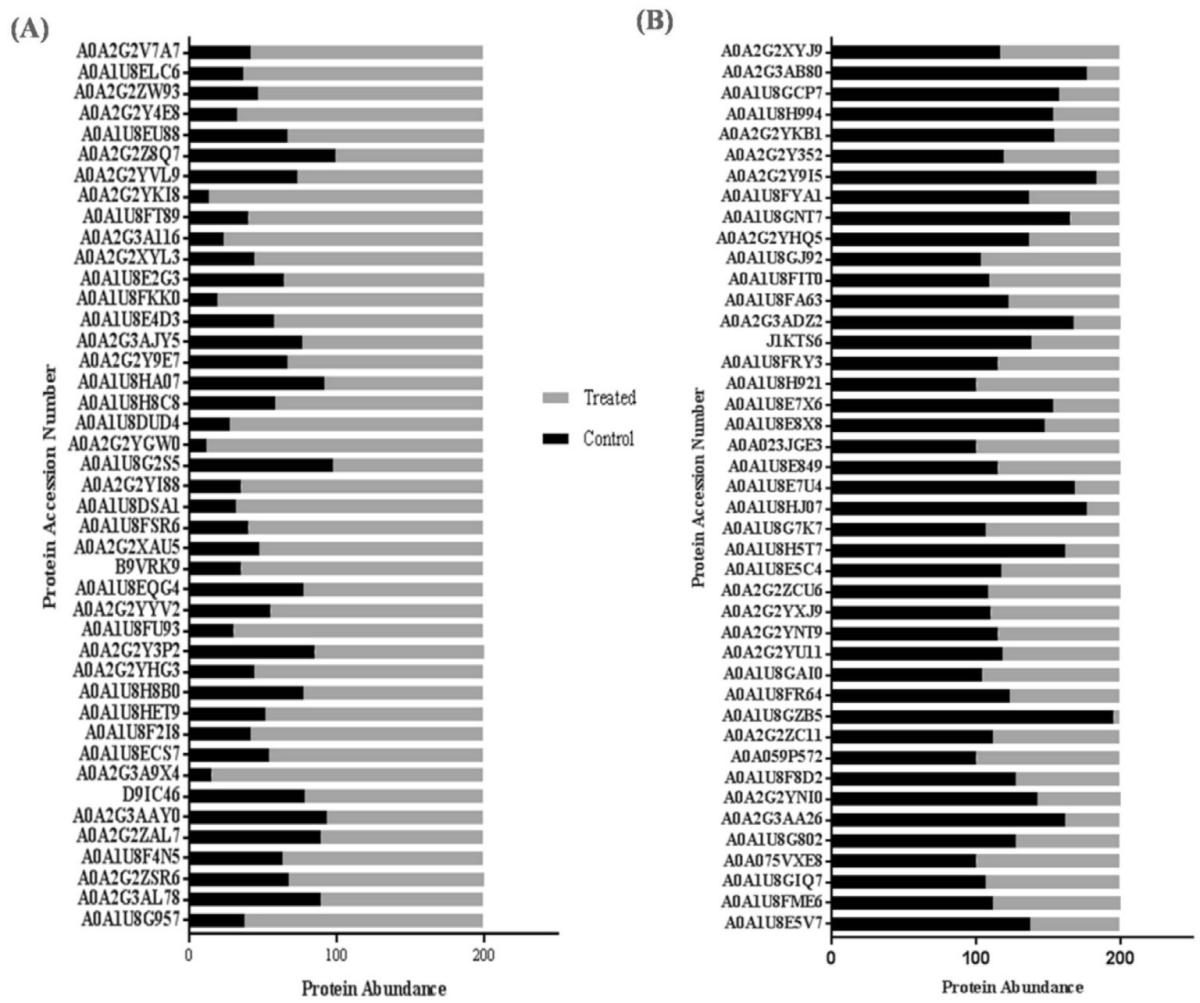
Drought induced apoplastic protein expression (A) Up-regulated proteins and (B) down-regulated proteins.

In the present study, differential expression of proteins was observed upon drought stress. Among 106 protein species identified, 43 proteins were up-regulated and 43 proteins were down-regulated in treated sample in comparison with control (Fig. 3). Twenty protein species were found to be uniquely identified in drought treated sample.

Drought induced apoplast proteome exhibited increased abundance in 10 proteins and decreased abundance in 19 proteins which were involved in diverse metabolic processes. This shows the negative effect of drought on various metabolic processes. Decreased expression levels of proteins involved in cell organisation can be implicated to depletion in cell organisation ability of plant cell under drought. Coping with a variety of abiotic stresses is highly dependent on up and down-regulation of proteins resulted from altered gene expression. Though most of the proteins were expressed under normal conditions, differential expression is often seen under stress conditions [8]. Imbalance in cellular redox metabolism under drought results in increased oxidative damage. To counterattack, plants produce several ROS scavenging enzymes. In our present study, among four peroxidases that were identified, one peroxidase (B9VRK9) was up-regulation where as three were down-regulated and there is a non-significant increase in the abundance of superoxide dimutase in treated sample. Kosova et al. [9], also reported increased abundance of ROS scavenging enzymes under cold in wheat. Drought induced chilli apoplast proteome revealed up-regulation of cell wall reprogramming proteins. Cell wall reprogramming was one of the important strategies of plant to withstand deleterious effects of stress [10].

**Fig. 4:**
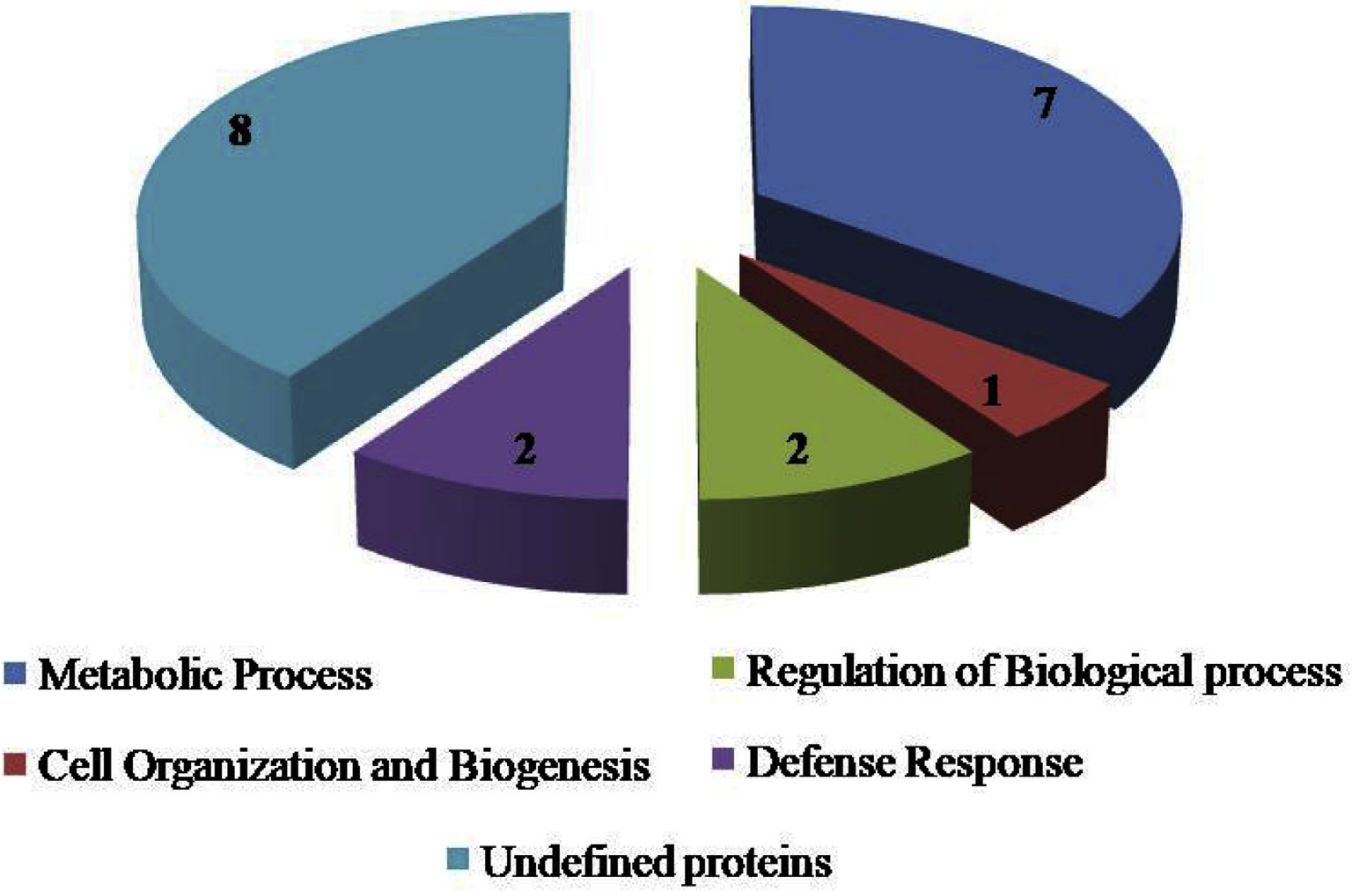
Functional Annotation of twenty Unique Proteins identified in drought treated chilli leaves.

Among 20 unique proteins identified in drought, seven proteins were related to metabolic processes, while two proteins were recognised to have role in regulation of biological process, two proteins were identified to take part in defence mechanism, one protein is known to play role in cell organisation and biogenesis and undefined (Fig. 4).

## 2 Experimental design, materials and methods

### 2.1 Plant material

Elite chilli genotype (S-10) seeds were procured from Horticultural Research Station, Lamfarm, Guntur, Andhra Pradesh. Seeds of S10 genotype were grown in black trays containing a mixture of peat and vermiculite (2:1 v/v) for 45 days followed by transplantation into pots (one plant/pot) and allowed for acclimatization for one week. Plants were grown in greenhouse under control conditions- 16 h light/8 h dark photoperiod at 27 °C during the day and 21 °C at night, and watered regularly.

### 2.2 Imposition of stress

Drought stress was imposed to plants using gravimetric method [11]. This method involves weighing pots twice a day followed by replenishing the water lost by evapotranspiration to maintain required field capacity (FC). Chilli plants were subjected to two water regimes viz., 100% FC (control), 40% FC (drought stress) for one week.

### 2.3 Apoplast protein extraction

Apoplastic proteins were extracted using the infiltration method described by (O'leary et al., [12]. All fresh green leaves were excised from plants and were washed in distilled water to remove cellular proteins from the cut ends. Leaves were dried and infiltrated using extraction buffer (0.1 M potassium phosphate buffer pH-7). Leaves were blotted gently, rolled carefully and loaded into 20ml syringe barrel. The syringe barrel was placed into centrifuge tubes. Apoplastic fluid was obtained at bottom of the tube after leaves were centrifuged at 1000×*g* for 15mins at 4 °C. The protein sample was immediately stored at −20 °C until further analysis.

### 2.4 Cytoplasmic contamination assay

Apoplastic fluid was tested for the presence of cytosolic contamination using Malate Dehydrogenase (MDH, EC 1.1.1.37) assay by comparing with whole leaf protein as a control according to method described by Alves et al., [13]. Apoplast protein extract was mixed with 50mM NADH, 0.2mM Tris-Hcl (pH 7.5) and 0.4mM oxoloacetate. Change in the absorbance at 340 nm was monitored over 3 min using UV/Visible Spectrophotometer (Eppendorf Biospectrometer Kinetic). To assess cytoplasmic contamination, total soluble proteins were extracted by using potassium phosphate buffer (pH-7). Leaves were homogenized in buffer and were centrifuged at 700×*g* for 10 mins at 4 °C (18), the supernatant was used for MDH enzyme assay. Cytoplasmic contamination was calculated as the percentage of MDH activity in the apoplast protein extract compared with activity in total leaf soluble protein extract.

### 2.5 Estimation of total phenolics (TP)

For the estimation of total phenolics, to 1ml of apoplastic extract 0.5ml of Folin-Ciocalteau reagent, 7.5ml ddH2O was added and incubated for 10 min at room temperature, and then 1.5ml of 20% sodium carbonate was added and incubated for 20 min at 400C. Solution was cooled and absorbance was recorded at 755 nm. Estimation of total phenolics (mg/g) was measured as described by Tohma et al., [14].

### 2.6 Estimation of phenylalanine ammonia lyase (PAL)

For the estimation of phenylalanine ammonia lyase content, to 0.3ml of apoplastic extract, 1.2ml of Tris buffer (25mM, pH-8.8) and 1.5 ml of l-phenylalanine (12mM) was added. The rate of conversion of l-phenylalanine to trans-cinnamic acid was determined at 290nm as described by Sri deepthi et al., [15].

### 2.7 Estimation of peroxidise activity

For the estimation of peroxidise activity, for 0.5 ml of apoplastic extract, 1.5 ml of pyrogallol solution (0.05 M) and 0.5ml of H2O2 was added. The change in absorbance was recorded at 430 nm for 3 min. POD activity was quantified according to the method described by Abhayashree et al. [16].

### 2.8 Estimation of catalase activity

For the estimation of catalase activity, to 40μl of apoplastic extract, 2.5ml of potassium phosphate buffer (50mM, pH-7) and 0.5ml of H2O2 were added. The rate of decomposition of H2O2 was determined at 240nm for 3 min. Catalase activity was quantified according to the method described by Huseynova et al. [17].

### 2.9 LC-MS analysis

#### 2.9.1 Sample preparation

Protein samples (50 μg) were reduced with 50 mM DTT at 60 °C for 1 h and the cysteine-groups were blocked using a 50 mM IAA solution at room temperature for 30 min. The protein samples were then subjected to trypsin digestion by adding trypsin in 1:30 ratio (Trypsin: Protein) at 37 °C in a dry bath for 16 hours. After trypsinization, samples were dried in speed vac and reconstituted in 20 μl of Milli-Q water with 0.1% formic acid and desalting was performed and then subjected to LC-MS.

#### 2.9.2 Proteome analysis

LC-MS analysis is performed in 1290 Infinity UHPLC system, 1260 infinity Nano HPLC with Chip cube, 6550 iFunnel Q-TOFs (Agilent technologies, USA) at Sophisticated Analytical Instrument Facility (SAIF), IIT Bombay. Samples were loaded in an analytical C18 column (PepMap RSLC C18 2 μm, 100 A × 50 cm). Mobile phase consists of solvent A: 0.1% FA in Milli-Q water, solvent B: 80:20 (ACN: Milli-Q water) + 0.1% FS. The raw LC-MS data was analyzed using Thermo Proteome Discoverer 2.2 software with Sequest-HT Uniport, capsicum annuum and plants databases.

#### 2.9.3 Statistical analysis

All the samples (for both assays and LC-MS analysis) were collected triplicate and data were analysed with One-Way ANOVA at 5% probability. Data were represented as Mean ± SD.
